# Emergency Department Pediatric Early Warning System for Identifying Resuscitation-Defined Critical Illness in Children: A Prospective Diagnostic Classification Study in Vietnam

**DOI:** 10.3390/pediatric18050131

**Published:** 2026-10-02

**Authors:** Duy Ngoc Le, Vu Duc Bui, Tung Viet Cao, Ly Ha Pham, Khiem Van Nguyen

**Affiliations:** 1 Vietnam National Children’s Hospital, Hanoi 10000, Vietnam; ngocduy@nch.gov.vn (D.N.L.); buiducvu93@gmail.com (V.D.B.); tungcv@nch.gov.vn (T.V.C.); 2 Department of Pediatrics, Faculty of Clinical Medicine, Vietnam University of Traditional Medicine, Hanoi 10000, Vietnam; phamhaly2012@gmail.com

**Keywords:** early warning score, clinical deterioration, child, pediatric emergency medicine, triage, Vietnam

## Abstract

Background/Objectives: Early recognition of children with severe physiological illness is essential in emergency care. The Emergency Department Pediatric Early Warning System score (ED-PEWS) has not been evaluated in Vietnam. We assessed how well ED-PEWS recorded at presentation identifies children who meet criteria for resuscitation-defined critical illness in a high-acuity pediatric emergency setting. Methods: This prospective observational diagnostic classification study was conducted from August to October 2022 in the emergency and resuscitation department of a national tertiary children’s hospital in Vietnam. Children aged 1 month to 15 years were enrolled according to a prespecified shift-based schedule. The outcome was resuscitation-defined critical illness within 24 h (cardiac arrest or bradycardia with poor perfusion requiring cardiopulmonary resuscitation). Because the timing of scoring relative to interventions and outcome was not recorded, the analysis was interpreted as concurrent classification rather than prediction. Results: Of 805 children (median age 19 months; 65.3% male), 156 (19.4%) met the outcome criteria. The median ED-PEWS was 32 (interquartile range, 24–43) in children with the outcome and 10 (8–12) in those without (*p* < 0.001). The area under the receiver operating characteristic curve was 0.952 (95% CI, 0.926–0.977). At the exploratory Youden-derived threshold of ED-PEWS ≥ 16, there were 143 true positives, 49 false positives, 13 false negatives, and 600 true negatives; sensitivity was 91.7% (95% CI, 86.2–95.5), specificity 92.4% (90.1–94.4), and negative predictive value 97.9% (96.4–98.9). Conclusions: In this selected high-acuity cohort, ED-PEWS showed strong concurrent discrimination for resuscitation-defined critical illness. Because the score and the outcome share physiological components and the threshold was derived and evaluated in the same dataset, these findings are exploratory and require multicenter external validation and comparison with existing triage systems.

## 1. Introduction

Children who become critically ill in the emergency department can be difficult to recognize early. Through compensatory mechanisms, a child may maintain a normal blood pressure and appear clinically stable until shortly before decompensation. If this phase is not recognized, the child may progress to respiratory failure, shock, bradycardia, or cardiac arrest and may require unplanned admission to intensive care [1,2].

Pediatric early warning scores (PEWS) were developed to provide a simple and reproducible assessment of illness severity in children. Early scores assessed behavior, circulation, and respiration [3]. The Bedside Paediatric Early Warning System showed that a simple score could discriminate critically ill from less severely ill children on hospital wards [4,5]. However, most of these scores were developed for inpatient wards rather than for the emergency department, where risk must be assessed at the time of presentation [6,7].

Several studies have tested PEWS in the emergency department, with mixed results that depend on the score and on the outcome studied [8,9,10]. Zachariasse et al. developed the ED-PEWS in a large European multicenter study. It comprises seven bedside items and was designed to stratify children into high, medium, and low urgency at triage; a high-urgency threshold above 15 points yielded a specificity of approximately 0.90 [11]. A later multicenter study in low- and middle-income countries found lower discrimination for high urgency (AUC approximately 0.62–0.80) and noted that the optimal threshold may vary between settings [12].

Two points are important for our study. First, ED-PEWS was developed to stratify children by urgency at triage, not to identify children who require resuscitation. Our outcome is more severe and less common, so our results cannot be compared directly with the original studies. Second, ED-PEWS has not been studied in Vietnam. We therefore conducted what is, to our knowledge, the first evaluation of ED-PEWS in Vietnam. We aimed to describe the types of illness and the ED-PEWS scores of children in a tertiary emergency and resuscitation department, and to measure how well ED-PEWS recorded at arrival discriminates children who met criteria for resuscitation-defined critical illness. This was framed as a diagnostic classification analysis rather than as a prognostic analysis of subsequent deterioration, because exact event timing was not available.

## 2. Materials and Methods

### 2.1. Study Design and Setting

This was a prospective observational diagnostic-classification study in the emergency and resuscitation department of a national tertiary children’s hospital in Vietnam, from 1 August to 31 October 2022. The hospital is a national tertiary referral center for children, and the department works as a high-acuity emergency and resuscitation unit. The cohort therefore represents children managed in this selected high-acuity setting and should not be interpreted as the denominator of all general pediatric emergency presentations. We followed the STARD statement for diagnostic accuracy [13] and the STROBE statement for observational studies [14]; the completed STARD 2015 checklist is provided as Supplementary File S1.

### 2.2. Participants and Sampling

Children aged 1 month to 15 years who came to the department with an emergency condition were eligible. We excluded children with incomplete data needed to calculate ED-PEWS, children whose 24-h course was unknown, and children who left the department before the study team could observe them. Because the department sees a large number of children each day, enrolment followed a prespecified fixed shift-based schedule in the parent study: one day or night shift was selected on alternate calendar days, and all eligible children presenting during each selected shift were enrolled. Children were not selectively recruited according to diagnosis or perceived severity. This was not consecutive sampling of the full emergency-department source population. The original dataset did not retain a complete log of all presentations during non-selected shifts, the number of children managed in lower-acuity areas, day–night balance in the analytic sample, referral status, triage categories, or repeat visits by the same child. The sample size was calculated for the primary aim of the parent study, based on the expected proportion of children admitted to hospital (0.56), with alpha = 0.01 (two-sided) and an absolute precision of 0.05, giving a minimum of 646 children. The present analysis was a secondary diagnostic-classification analysis that included all enrolled children who had a complete ED-PEWS and a known 24-h outcome (complete-case analysis). No separate sample-size calculation was made for the ROC analysis; the precision of diagnostic estimates is reflected by confidence intervals based on the observed 156 children with and 649 without the outcome.

### 2.3. ED-PEWS Assessment

ED-PEWS was recorded by trained study staff when the child arrived, using a standardized study form and the method described by Zachariasse et al. [11]. The score uses seven bedside items: age group, altered consciousness, use of accessory respiratory muscles, respiratory rate, oxygen saturation, heart rate, and prolonged capillary refill time. The total score ranges from 0 to 68 points; higher scores indicate greater physiological derangement. The score was collected for research only and was not embedded in a treatment protocol. Whether the treating team saw the score, whether individual physiological measurements were obtained before or after oxygen, ventilation, fluids, or vasoactive treatment, and the exact time of scoring in relation to the first emergency treatment were not recorded. The analytic dataset did not retain the number and professional background of individual assessors, detailed training records, component-level missingness before complete-case selection, or procedures for resolving uncertain observations. Formal inter-rater reliability testing was not performed.

### 2.4. Outcome

The outcome was resuscitation-defined critical illness within 24 h of study entry, defined as cardiac arrest (no central pulse) or bradycardia (heart rate below 60 beats per minute) with poor perfusion (mottled or cold skin, pallor, agitation, lethargy, or coma) that needed cardiopulmonary resuscitation [2]. The need for cardiopulmonary resuscitation was determined by the treating clinical team according to standard pediatric resuscitation practice and documented in the prospective study record and clinical record. The analytic dataset did not reliably separate cardiac arrest from bradycardia with poor perfusion, and return of spontaneous circulation or death was not consistently available for analysis. The original dataset did not record whether outcome assessors were blinded to ED-PEWS values or to the study hypothesis; therefore, formal blinding of outcome ascertainment could not be confirmed. The exact time of the outcome in relation to arrival, first emergency interventions, and ED-PEWS scoring was not recorded. For this reason, the analysis shows how well ED-PEWS classifies children who met criteria for this critical state within 24 h, rather than predicting a clearly later event.

### 2.5. Statistical Analysis

Categorical variables are shown as numbers and percentages and compared with the chi-square or Fisher exact test. Continuous variables that were not normally distributed are shown as medians with interquartile ranges (IQRs) and compared with the Mann–Whitney U or Kruskal–Wallis test. We measured how well ED-PEWS separates children with and without the outcome using the AUC, with its 95% confidence interval (CI) estimated by the DeLong method [15]. The best threshold was found as the score with the highest Youden index (sensitivity + specificity − 1); we describe this as an exploratory, data-derived statistical threshold. Because ED-PEWS is a whole-number score in practice, the value of 15.5 is reported as ED-PEWS ≥ 16. For selected thresholds, we report underlying classification counts (true positives, false positives, false negatives, and true negatives), sensitivity, specificity, predictive values, and positive and negative likelihood ratios (LR+, LR−). Exact binomial 95% CIs were calculated for sensitivity, specificity, positive predictive value, and negative predictive value. CIs for LR+ and LR− were calculated using the log method. Because ED-PEWS gives an ordinal score rather than a probability, calibration was not assessed; we report discrimination and threshold-based classification only. The primary analyses (descriptive statistics, group comparisons, ROC analysis, AUC with DeLong CI, and Youden index) were performed on the complete-case participant-level dataset (*n* = 805) at the time of the original study. The participant-level dataset is held by the parent study at the study hospital and could not be accessed by the authors for reanalysis during this revision. For the revision, threshold-specific classification counts were therefore derived from the original analysis output (sensitivity and specificity at each ED-PEWS cut-off, applied to the 156 children with and 649 children without the outcome), and exact binomial and log-method CIs were calculated from these counts. Internal validation of the data-derived threshold (for example, by bootstrapping or cross-validation) was not performed, because such resampling requires the participant-level scores and outcomes. Analyses used SPSS version 26.0 (IBM Corp., Armonk, NY, USA) and Microsoft Excel; a two-sided *p* < 0.05 was considered significant.

### 2.6. Ethics

The study protocol was approved by the Institutional Review Board (Biomedical Research Ethics Committee) of Vietnam National Children’s Hospital (IRB-VN01037/IRB00011976/FWA00028418; approval No. 1555/BVNTW-HĐĐĐ, dated 18 July 2022) as part of the parent study “Application of the Emergency Department Pediatric Early Warning Score (ED-PEWS) at the Emergency and Poison Control Department of Vietnam National Children’s Hospital”; the present analysis was performed within the scope of this approval. Data collection began after approval was granted. The study was carried out in accordance with the Declaration of Helsinki (2013 revision). Written informed consent was obtained from parents or legal guardians. Data were de-identified for analysis.

## 3. Results

### 3.1. Participants and Types of Illness

**Figure 1:**
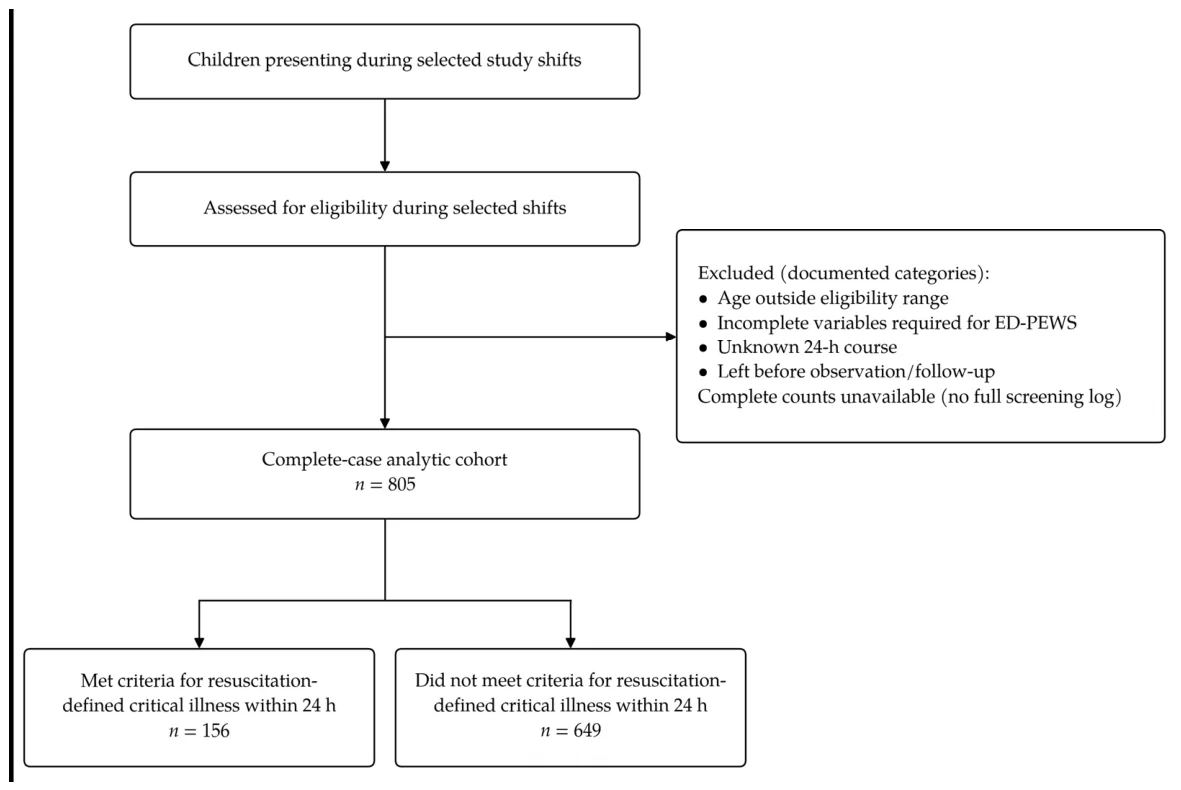
Participant flow summary based on available data. A complete screening log of all emergency department presentations was not available; therefore, the numbers of children screened and excluded could not be reported.

We included 805 children (median age 19 months [IQR, 7–58]; 526 [65.3%] male). Resuscitation-defined critical illness occurred in 156 children (19.4%). This high frequency reflects the selected high-acuity emergency and resuscitation-unit case spectrum rather than the full denominator of all pediatric emergency presentations. A complete screening log of all emergency department presentations during the study period was not available; therefore, Figure 1 is presented as a participant flow summary based on available data, from enrolled children to the complete-case analytic cohort, rather than as a complete STARD participant flow diagram. Age, sex, and residence did not differ significantly between children who did and did not meet the outcome criteria (Table 1). Respiratory disease was the most common group (513 children; 63.7%), followed by allergic and immunologic disorders (86; 10.7%), neurological disease (61; 7.6%), cardiovascular disease (38; 4.7%), and infectious disease (31; 3.9%). Disease groups were assigned from the final clinical diagnosis recorded in the parent study and treated as the primary mutually exclusive diagnostic category for this analysis. The local classification separated ‘infectious disease’ (mainly sepsis and bacterial infections) from ‘communicable disease’ (notifiable transmissible infections). The most common single diagnoses were pneumonia (32.4%), severe acute bronchiolitis (10.8%), and multisystem inflammatory syndrome in children (9.7%). The proportion reaching the outcome within each group was highest for infectious disease (38.7%), allergic and immunologic disorders (32.6%), and cardiovascular disease (26.3%) (Table 2).

### 3.2. ED-PEWS Scores

The median ED-PEWS in the whole cohort was 12 (IQR, 8–15; range 0–68). Scores were substantially higher in children who met the outcome criteria than in those who did not (median 32 [IQR, 24–43] vs. 10 [IQR, 8–12]; *p* < 0.001) (Table 3).

### 3.3. Discrimination and Thresholds

**Figure 2:**
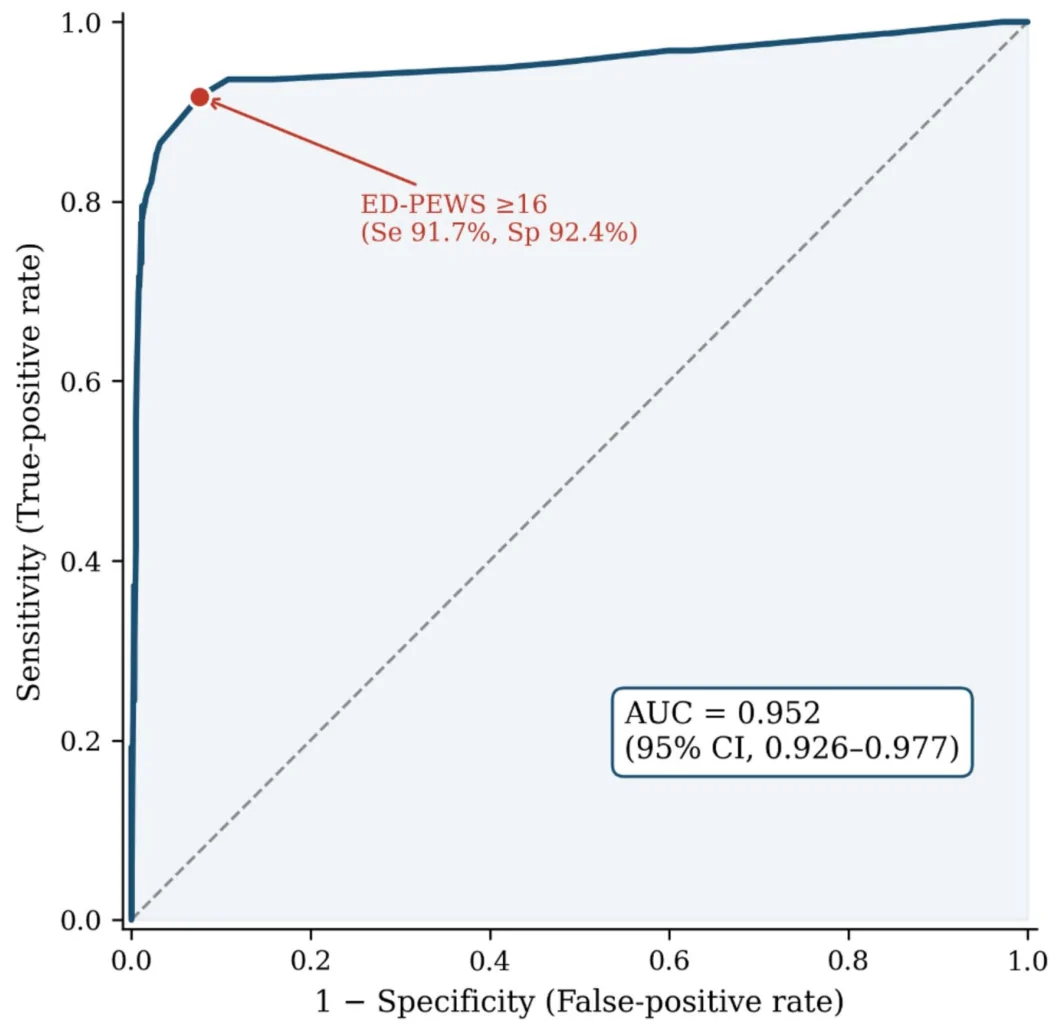
ROC curve of ED-PEWS for resuscitation-defined critical illness. Area under the curve 0.952 (95% CI, 0.926–0.977). The marked point is the exploratory Youden-derived threshold ED-PEWS ≥ 16 (sensitivity 91.7%, specificity 92.4%).

ED-PEWS showed high discrimination for resuscitation-defined critical illness, with an AUC of 0.952 (95% CI, 0.926–0.977) (Figure 2). The Youden index was highest at ED-PEWS ≥ 16. At this exploratory threshold, there were 143 true positives, 49 false positives, 13 false negatives, and 600 true negatives. Sensitivity was 91.7% (95% CI, 86.2–95.5), specificity 92.4% (95% CI, 90.1–94.4), positive predictive value 74.5% (95% CI, 67.7–80.5), negative predictive value 97.9% (95% CI, 96.4–98.9), LR+ 12.1 (95% CI, 9.2–16.0), and LR− 0.09 (95% CI, 0.05–0.15). Higher thresholds yielded higher specificity but lower sensitivity. Classification counts and 95% CIs for selected thresholds are shown in Table 4.

## 4. Discussion

In this prospective diagnostic-classification study of 805 children at a Vietnamese tertiary emergency and resuscitation unit, ED-PEWS measured at arrival strongly discriminated children who met criteria for resuscitation-defined critical illness (AUC 0.952). Scores were substantially higher in children who met the outcome criteria. To our knowledge, these are the first ED-PEWS data from Vietnam. However, the findings should be interpreted as concurrent discrimination/classification of severe physiological illness, not as evidence that ED-PEWS predicts subsequent deterioration.

These results should be read together with the original purpose of ED-PEWS, which was to stratify children by urgency at triage rather than to identify children requiring resuscitation [11]. Differences in AUC across studies should not be interpreted without considering patient spectrum, clinical setting, sampling strategy, outcome definition, and timing of outcome assessment. In low- and middle-income settings, ED-PEWS discriminated high-urgency children with an AUC of approximately 0.62–0.80 [12], and earlier emergency-department studies reported AUCs of approximately 0.60–0.82 for intensive care admission [8]. The higher AUC observed in our study does not indicate superior performance of the tool. It reflects a different and more severe outcome, a selected high-acuity referral/resuscitation case mix, and a closer link between the score and the outcome. ED-PEWS includes heart rate, level of consciousness, oxygen saturation, respiratory effort, and capillary refill time, while the outcome includes cardiac arrest or bradycardia with poor perfusion requiring cardiopulmonary resuscitation. The score and the outcome therefore share several physiological components. This overlap (criterion contamination) may have increased the apparent discriminatory performance, and the high AUC is best understood as the score identifying children who were already, or almost, critically ill rather than predicting a later independent event.

We describe ED-PEWS ≥ 16 as the Youden-optimal exploratory statistical threshold, not as a validated threshold or the best clinical threshold. Although ED-PEWS ≥ 16 maximized the Youden index in this cohort, the selection of a clinically actionable threshold should also consider the intended rule-in or rule-out purpose, outcome prevalence, resource implications, and the relative consequences of false-negative and false-positive classifications [16]. The Youden index gives equal weight to sensitivity and specificity, but in pediatric emergency care the clinical consequences of false-negative and false-positive classifications are not equivalent. At ED-PEWS ≥ 16, 13 children were false negatives and 49 were false positives. A false-negative result may delay escalation for a child with established or evolving critical illness, whereas a false-positive result may increase monitoring burden, staff workload, or unnecessary resuscitation-area use. The clinically preferred threshold may therefore differ according to local resources and the intended purpose of the score, and should be evaluated in future decision-analytic or implementation studies.

Our statistical threshold (≥16) is numerically close to the high-urgency threshold above 15 points in the original ED-PEWS development study [11]. The numerical similarity is notable, but it should not be interpreted as validation, because the target outcomes, case mix, patient spectrum, sampling strategy, and timing of outcome assessment differed substantially. The original ED-PEWS studies evaluated triage urgency or later need for higher-level care, whereas this study used a narrower resuscitation-defined outcome that overlaps with several score components. The present study therefore extends the ED-PEWS literature by describing its performance in a Vietnamese national pediatric referral setting, but it does not establish clinical utility, transportability, or a universally applicable threshold.

Respiratory disease was the most common group in both the whole cohort and the critical-illness group. This fits with respiratory failure being a common final pathway in children, while infectious and allergic or immunologic conditions had the highest within-group rates. The high overall outcome frequency and high within-group rates in some diagnostic categories probably reflect the selected high-acuity emergency/resuscitation setting and referral case spectrum. We did not estimate performance within individual disease or triage groups, because the numbers were small, triage category data were not available, and the estimates would be unstable.

ED-PEWS is not intended to replace established emergency triage algorithms, such as the World Health Organization Emergency Triage Assessment and Treatment (ETAT) guidelines or local triage protocols [1]. ETAT uses emergency and priority signs to allocate children to immediate treatment, rapid assessment, or the routine queue, whereas ED-PEWS provides a graded physiological score. Future studies in low- and middle-income settings should therefore compare ED-PEWS with ETAT in the same children against a common reference standard, and evaluate a sequential approach in which ETAT emergency signs trigger immediate resuscitation and ED-PEWS is used to stratify children without emergency signs for monitoring and escalation, with time to first intervention, under- and over-triage, and staff workload as workflow outcomes.

The strengths of this study are its prospective design, a relatively large cohort with 156 outcome events, a simple bedside score, and data from a high-acuity pediatric emergency setting that has not been studied before.

## 5. Limitations of the Study

This study has several limitations. It was conducted in a single high-acuity tertiary center, so the mix of patients and the high outcome rate (19.4%) may not apply to general or lower-acuity emergency departments. The shift-based sampling strategy may have introduced selection or spectrum bias if acuity, referral patterns, staffing, or case mix differed between selected and non-selected shifts, and a complete screening log of all emergency department presentations was not available. We could not compare day and night shifts, quantify lower-acuity denominators, identify referral status or triage categories, or confirm whether each record was a different child or a repeat visit. The outcome was a severe, resuscitation-defined state and did not include other outcomes such as intensive care admission, death, or length of stay. Its components (cardiac arrest versus bradycardia with poor perfusion), return of spontaneous circulation, and death were not reliably available for separate analysis. The exact time of the outcome in relation to arrival, first treatment, and ED-PEWS assessment was not recorded. Formal blinding of outcome assessors to ED-PEWS values was not documented, and formal inter-rater reliability testing for ED-PEWS was not performed. We also could not reconstruct component-level missingness or verify whether physiological measurements were obtained before or after initial resuscitation interventions. The overlap between the score and the outcome (criterion contamination) may make the AUC look higher than the true predictive value for a later independent outcome. The ≥16 threshold was found and evaluated in the same dataset using the Youden index, so it may be over-optimistic; it was not tested by bootstrapping or cross-validation or in another center. We could not assess calibration, we did not compare ED-PEWS with ETAT or the current local triage system, and we did not measure clinical utility using decision-curve or impact analysis. Future studies should test ED-PEWS in several Vietnamese centers, record the timing of deterioration and interventions, compare it with current triage, assess calibration and inter-rater reliability, report outcome components separately, and evaluate the clinical consequences of different thresholds.

## 6. Conclusions

In this selected, high-acuity Vietnamese cohort, ED-PEWS measured at arrival showed strong concurrent discrimination for children who met criteria for resuscitation-defined critical illness, and an exploratory statistical threshold of ED-PEWS ≥ 16 identified a high-risk group. Because the score and the outcome share several physiological components, the exact temporal sequence was not recorded, the threshold was derived and evaluated in the same dataset, and ED-PEWS was not compared with current triage or decision-analytic approaches, these findings should be treated as exploratory. Multicenter external validation, broader outcomes, comparison with existing triage systems, and evaluation of net clinical benefit are needed before ED-PEWS thresholds are used to guide escalation of care.

## Figures and Tables

**Table 1:** Characteristics of children by outcome (*N* = 805).

Characteristic	Critical Illness (*n* = 156), *n* (%)	No Critical Illness (*n* = 649), *n* (%)	*p*
Age group			0.077
≥1 to <3 months	20 (12.8)	65 (10.0)	
≥3 to <12 months	40 (25.6)	149 (23.0)	
≥1 to <4 years	43 (27.6)	258 (39.8)	
≥4 to ≤12 years	49 (31.4)	166 (25.6)	
>12 years	4 (2.6)	11 (1.7)	
Sex			0.717
Male	100 (64.1)	426 (65.6)	
Female	56 (35.9)	223 (34.4)	
Residence			0.512
Urban	75 (48.1)	331 (51.0)	
Rural	81 (51.9)	318 (49.0)	

Overall median age was 19 months (IQR, 7–58). *p*-values were calculated from the reported counts using Pearson chi-square tests (for age group, 1 of 10 cells had an expected count <5). Between-group comparisons were descriptive and were not the primary focus of the analysis.

**Table 2:** Disease groups and resuscitation-defined critical illness within 24 h.

Disease Group	Total, *n* (%)	Critical Illness, *n*	No Critical Illness, *n*	Within-Group Rate, %
Respiratory	513 (63.7)	92	421	17.9
Allergic and immunologic disorders	86 (10.7)	28	58	32.6
Neurological	61 (7.6)	10	51	16.4
Cardiovascular	38 (4.7)	10	28	26.3
Infectious disease	31 (3.9)	12	19	38.7
Gastrointestinal	16 (2.0)	0	16	0.0
Communicable disease	15 (1.9)	1	14	6.7
Oncological	14 (1.7)	0	14	0.0
Hematological	9 (1.1)	1	8	11.1
Endocrine	7 (0.9)	1	6	14.3
Hepatobiliary	6 (0.7)	0	6	0.0
Renal and urinary	5 (0.6)	1	4	20.0
Nutritional	4 (0.5)	0	4	0.0
Total	805 (100)	156	649	19.4

Resuscitation-defined critical illness: cardiac arrest or bradycardia with poor perfusion requiring cardiopulmonary resuscitation within 24 h of study entry. Within-group rate = number of children with resuscitation-defined critical illness divided by the group total. Disease groups followed the local classification used in the parent study and were treated as the primary mutually exclusive diagnostic category for this analysis. “Infectious disease” refers mainly to sepsis and bacterial infections, and “communicable disease” to notifiable transmissible infections.

**Table 3:** ED-PEWS scores by outcome.

Group	*n*	Median	IQR	Minimum	Maximum	*p*
Whole cohort	805	12	8–15	0	68	—
Critical illness	156	32	24–43	6	68	<0.001
No critical illness	649	10	8–12	0	46	Reference

ED-PEWS: Emergency Department Pediatric Early Warning System score; IQR: interquartile range. Comparison by Mann–Whitney U test.

**Table 4:** Performance of ED-PEWS at selected thresholds for resuscitation-defined critical illness.

Threshold	TP	FP	FN	TN	Sensitivity, % (95% CI)	Specificity, % (95% CI)	PPV, % (95% CI)	NPV, % (95% CI)	LR+ (95% CI)	LR− (95% CI)
≥13	146	102	10	547	93.6 (88.5–96.9)	84.3 (81.3–87.0)	58.9 (52.5–65.1)	98.2 (96.7–99.1)	5.95 (4.96–7.15)	0.08 (0.04–0.14)
≥14	146	88	10	561	93.6 (88.5–96.9)	86.4 (83.6–89.0)	62.4 (55.8–68.6)	98.2 (96.8–99.2)	6.90 (5.66–8.42)	0.07 (0.04–0.14)
≥15	146	70	10	579	93.6 (88.5–96.9)	89.2 (86.6–91.5)	67.6 (60.9–73.8)	98.3 (96.9–99.2)	8.68 (6.93–10.87)	0.07 (0.04–0.13)
≥16 *	143	49	13	600	91.7 (86.2–95.5)	92.4 (90.1–94.4)	74.5 (67.7–80.5)	97.9 (96.4–98.9)	12.14 (9.24–15.96)	0.09 (0.05–0.15)
≥17	135	21	21	628	86.5 (80.2–91.5)	96.8 (95.1–98.0)	86.5 (80.2–91.5)	96.8 (95.1–98.0)	26.74 (17.48–40.92)	0.14 (0.09–0.21)
≥18	133	18	23	631	85.3 (78.7–90.4)	97.2 (95.7–98.3)	88.1 (81.8–92.8)	96.5 (94.8–97.8)	30.74 (19.40–48.70)	0.15 (0.10–0.22)
≥20	126	11	30	638	80.8 (73.7–86.6)	98.3 (97.0–99.2)	92.0 (86.1–95.9)	95.5 (93.7–96.9)	47.65 (26.39–86.05)	0.20 (0.14–0.27)

* Youden-derived exploratory statistical threshold. Because ED-PEWS is an integer score, the Youden-derived value of 15.5 is operationalized as ED-PEWS ≥ 16. The ≥16 threshold was selected and evaluated in the same cohort and should be considered exploratory. Sensitivity, specificity, PPV, and NPV: exact binomial (Clopper–Pearson) 95% CIs; LR+ and LR−: log-method 95% CIs. TP: true positives; FP: false positives; FN: false negatives; TN: true negatives; PPV: positive predictive value; NPV: negative predictive value; LR+: positive likelihood ratio; LR−: negative likelihood ratio; CI: confidence interval.

## Data Availability

The participant-level data underlying this study are held by the parent study at Vietnam National Children’s Hospital and are not publicly available because of institutional data-governance and confidentiality requirements; they were not accessible to the authors for reanalysis during revision. Aggregated data supporting the findings are presented in the article (Table 1, Table 2, Table 3 and Table 4 and Figure 1 and Figure 2). Requests for access to de-identified participant-level data may be sent to the corresponding author, who will forward them to the data custodian; access is subject to approval by the hospital’s Institutional Review Board.

## Supplementary Materials

The following supporting information can be downloaded at: https://www.mdpi.com/article/10.3390/pediatric18050131/s1, File S1: STARD 2015 checklist.
