# Supplementary material for: Emergency Department Pediatric Early Warning System for Identifying Resuscitation-Defined Critical Illness in Children: A Prospective Diagnostic Classification Study in Vietnam
Source: Pediatr Rep. 2026 Oct 2;18(5):131. doi: 10.3390/pediatric18050131 (PMC13708330; doi:10.3390/pediatric18050131)
Supplement: Supplementary file 1 [file pediatrrep-18-00131-s001.zip › pediatrrep-4550552-supplementary.pdf]

Supplementary File S1. STARD 2015 checklist

Manuscript: Emergency department pediatric early warning system for identifying resuscitation-defined critical illness in children: a prospective diagnostic classification study in Vietnam (pediatrrep-4550552). Line numbers refer to the clean revised manuscript.

Section & topic	No.	STARD 2015 item	Reported in (section; line numbers of clean revised manuscript)
Title or abstract	1	Identification as a study of diagnostic accuracy using at least one measure of accuracy (such as sensitivity, specificity, predictive values, or AUC)	Title (“diagnostic classification study”); Abstract, lines 5–27 (sensitivity, specificity, NPV, AUC)
Abstract	2	Structured summary of study design, methods, results, and conclusions	Abstract, lines 5–27
Introduction	3	Scientific and clinical background, including the intended use and clinical role of the index test	Introduction, lines 32–54
	4	Study objectives and hypotheses	Introduction, final paragraph (lines 54–63)
Methods – Study design	5	Whether data collection was planned before the index test and reference standard were performed (prospective study) or after (retrospective study)	Study design and setting, lines 65–73 (prospective data collection within the parent study)
Participants	6	Eligibility criteria	Participants and sampling, lines 74–78
	7	On what basis potentially eligible participants were identified	Lines 74–78 (presentation to the department with an emergency condition)
	8	Where and when potentially eligible participants were identified (setting, location and dates)	Lines 65–71 (national tertiary children’s hospital, Vietnam; 1 August–31 October 2022)
	9	Whether participants formed a consecutive, random or convenience series	Lines 78–86 (prespecified shift-based schedule; all eligible children in selected shifts; explicitly not consecutive sampling)
Test methods	10a	Index test, in sufficient detail to allow replication	ED-PEWS assessment, lines 96–110 (seven items, 0–68 points, method of Zachariasse et al. [10])
	10b	Reference standard, in sufficient detail to allow replication	Outcome, lines 111–126
	11	Rationale for choosing the reference standard (if alternatives exist)	Introduction lines 50–62; Outcome lines 111–118; Discussion (outcome differs from triage urgency; criterion contamination)
	12a	Definition of and rationale for test positivity cut-offs or result categories of the index test, distinguishing pre-specified from exploratory	Statistical analysis, lines 134–138 (Youden-derived ≥16, explicitly exploratory); Table 4 footnote
	12b	Definition of and rationale for test positivity cut-offs or result categories of the reference standard, distinguishing pre-specified from exploratory	Outcome, lines 111–118 (pre-specified binary definition: cardiac arrest or bradycardia with poor perfusion requiring CPR)
	13a	Whether clinical information and reference standard results were available to the performers/readers of the index test	ED-PEWS assessment, lines 96–110 (scored at presentation by study staff; timing relative to outcome not recorded – reported as limitation)
	13b	Whether clinical information and index test results were available to the assessors of the reference standard	Outcome, lines 118–123 (blinding not recorded; could not be confirmed); Limitations
Analysis	14	Methods for estimating or comparing measures of diagnostic accuracy	Statistical analysis, lines 127–156
	15	How indeterminate index test or reference standard results were handled	Not applicable: ED-PEWS yields a numerical score and the reference standard is binary; records with incomplete data were excluded (lines 76–78, 90–93)

Section & topic	No.	STARD 2015 item	Reported in (section; line numbers of clean revised manuscript)
	16	How missing data on the index test and reference standard were handled	Complete-case analysis, lines 76–78 and 90–93; component-level missingness not retained (lines 106–109)
	17	Any analyses of variability in diagnostic accuracy, distinguishing pre-specified from exploratory	Not performed; reason given in Discussion (lines 268–275: small subgroup numbers, no triage data)
	18	Intended sample size and how it was determined	Lines 86–95 (calculated for parent study; no separate calculation for ROC analysis)
Results – Participants	19	Flow of participants, using a diagram	Figure 1 (participant flow summary based on available data; screening counts unavailable – lines 175–178, 191)
	20	Baseline demographic and clinical characteristics of participants	Results lines 169–179; Table 1
	21a	Distribution of severity of disease in those with the target condition	Table 2 (disease groups), Table 3 (ED-PEWS distribution); outcome components not separable (Outcome lines 118–120; Limitations)
	21b	Distribution of alternative diagnoses in those without the target condition	Table 2; Results lines 179–190
	22	Time interval and any clinical interventions between index test and reference standard	Not recorded; reported explicitly as a limitation (ED-PEWS assessment lines 102–106; Outcome lines 123–126; Limitations)
Test results	23	Cross tabulation of the index test results (or their distribution) by the results of the reference standard	Table 3 (score distribution by outcome); Table 4 (TP, FP, FN, TN at each threshold); Results lines 207–216
	24	Estimates of diagnostic accuracy and their precision (such as 95% confidence intervals)	Results lines 207–216; Table 4; Figure 2
	25	Any adverse events from performing the index test or the reference standard	Not applicable: ED-PEWS is a non-invasive bedside observation and the reference standard was determined during routine clinical care; adverse events were not separately recorded
Discussion	26	Study limitations, including sources of potential bias, statistical uncertainty, and generalisability	Limitations of the Study, lines 290–318
	27	Implications for practice, including the intended use and clinical role of the index test	Discussion lines 244–286; Conclusions lines 319–329
Other information	28	Registration number and name of registry	Not registered (observational diagnostic study)
	29	Where the full study protocol can be accessed	The IRB-approved protocol of the parent study is not publicly available; it may be requested from the corresponding author (Data Availability Statement, lines 339–346)
	30	Sources of funding and other support; role of funders	Funding statement, line 348 (no external funding)

Checklist items from: Bossuyt PM, Reitsma JB, Bruns DE, et al. STARD 2015: an updated list of essential items for reporting diagnostic accuracy studies. BMJ. 2015;351:h5527.
